# A meta-analysis of leadership and intrinsic motivation: Examining relative importance and moderators

**DOI:** 10.3389/fpsyg.2022.941161

**Published:** 2022-08-12

**Authors:** Hanbing Xue, Yifei Luo, Yuxiang Luan, Nan Wang

**Affiliations:** ^1^School of Labor and Human Resources, Renmin University of China, Beijing, China; ^2^School of Business, North Minzu University, Yinchuan, China

**Keywords:** leadership, intrinsic motivation, self-determination theory, meta-analysis, transformational leadership

## Abstract

This paper provides the first meta-analytic examination of the relationship between leadership and followers' intrinsic motivation. In particular, we examined 6 leadership variables (transformational, ethical, leader-member exchange, servant, empowering, and abusive supervision) using data from 50 independent samples and 21,873 participants. We found that transformational leadership, ethical leadership, leader-member exchange (LMX), servant leadership, and empowering leadership were positively related to intrinsic motivation, whereas abusive supervision was negatively linked to intrinsic motivation. Although these leadership styles were associated with intrinsic motivation, they varied considerably in their relative importance. Empowering, ethical, and servant leadership emerged as the more important contributors to intrinsic motivation than transformational leadership. LMX showed a similar contribution with transformational leadership to intrinsic motivation. Effectiveness of leadership styles in relation to intrinsic motivation varied by power distance, publication year, and journal quality. Drawing on our findings, we discuss the theoretical and practice implications.

## Introduction

About a half-century ago, Deci (1971) found external reward would undermine intrinsic motivation and published his well-known paper about intrinsic motivation. He aroused people's great interest (intrinsic motivation) in studying intrinsic motivation. Since then, intrinsic motivation, which refers to doing something because it is inherently interesting or enjoyable (Ryan and Deci, 2000a; Sheldon and Prentice, 2019), has drawn so much academic attention. In the workplace, when employees are intrinsically motivated, they are likely to achieve high-quality performance (Deci et al., 2017). For example, meta-analyses provided solid evidence that intrinsic motivation is strongly and positively related to creativity (de Jesus et al., 2013) and work performance (Cerasoli et al., 2014). Besides, experiments showed that intrinsic motivation influences individuals' psychological wellbeing (Burton et al., 2006).

Given the importance of intrinsic motivation in work, not surprisingly, scholars, and managers are seeking the answers to the following question: what factors could influence intrinsic motivation? Drawing on self-determination theory (SDT) (Deci and Ryan, 2000; Gagné and Deci, 2005), motivation would be influenced by contextual factors. As such, scholars try to detect the contextual antecedents of intrinsic motivation. Leadership is an important factor that would influence employees' wellbeing (Salas-Vallina and Alegre, 2018) and intrinsic motivation (Deci et al., 2017). Although fruitful evidence between leadership and intrinsic motivation has been accumulated, some unsolved issues still exist.

First, true population correlations between leadership styles and intrinsic motivation have not been evaluated yet. Primary studies would suffer from statistical artifacts and thereby conclude different correlations of interest (Hunter and Schmidt, 2004). For example, for the association between transformational leadership and intrinsic motivation, Nguyen et al. (2022) found a small magnitude of the effect size (*r* = 0.03), while Al Harbi et al. (2019) found a medium one (*r* = 0.30). Fortunately, meta-analysis methodology could help us to correct the statistical artifacts and estimate the true population correlations of interest. As such, in the current study, we will evaluate the links between intrinsic motivation and various leadership styles (i.e., transformational, ethical, servant, empowering, LMX, and abusive supervision). By doing so, we seek to contribute to leadership and motivation literature.

Second, the relative importance of leadership to intrinsic motivation is not clear. Following early meta-analyses (Hoch et al., 2018; Lee et al., 2020a,b), we will compare the relative importance of transformational leadership and other types of leadership in the current study. This effort would not only enrich our understanding of the relationship between leadership and intrinsic motivation but also provide meaningful management suggestions for managers. For instance, to increase followers' intrinsic motivation, managers can use suitable leadership according to our meta-analytic results.

Finally, the potential moderators of the relationship between leadership and intrinsic motivation have not been detected yet. For instance, a previous meta-analysis (Lee et al., 2020b) found that correlations of interest are higher when using a common source research design. In this study, we will detect five potential moderators. That is publication year, source (common source vs. non-common source), power distance, individualism, and quality of the journal.

## Theoretical background and hypotheses development

### Leadership and intrinsic motivation

In the current study, we research the links between six types of leadership and intrinsic motivation. We focus on these six types of leadership (rather than other leadership) for three reasons. First, a recent review of SDT (Deci et al., 2017) suggested that transformational leadership would influence their followers' intrinsic motivation. As such, transformational leadership should be taken into consideration. Deci et al. (2017) also suggested researching other types of leadership and their relations with motivation. Second, based on leadership literature, meta-analyses about leadership and creativity (Lee et al., 2020a) and engagement (Li et al., 2021) consider these types of leadership. That is to say, these leadership styles capture scholars' research interest to some extent. Finally, to accurately estimate the links between leadership and intrinsic motivation, the leadership style should include more than 3 primary studies. As such, we include leadership styles that have more than 3 primary studies. We present their definitions in Table 1.

**Table 1:** Leadership definition.

**Leadership**	**Definition**
Transformational leadership	Transformational leadership refers to “the leader moving the follower beyond immediate self-interests through idealized influence (charisma), inspiration, intellectual stimulation, or individualized consideration” ((Bass, 1999), p. 11).
Servant leadership	Eva et al. (2019) defined servant leadership as leadership that “(1) other-oriented approach to leadership (2) manifested through one-on-one prioritizing of follower individual needs and interests, (3) and outward reorienting of their concern for self toward concern for other within the organization and the larger community” (Eva et al., 2019, p. 114).
LMX	LMX reflects the exchange quality between leaders and their followers. “Low LMX relationships are characterized by economic exchange based on formally agreed on, immediate, and balanced reciprocation of tangible assets, such as employment contracts focusing on pay for performance; high-LMX relationships increasingly engender feelings of mutual obligation and reciprocity” (Dulebohn et al., 2012, p. 1,717).
Empowering leadership	Empowering leadership is “the process of influencing subordinates through power sharing, motivation support, and development support with intent to promote their experience of self-reliance, motivation, and capability to work autonomously within the boundaries of overall organizational goals and strategies” (Amundsen and Martinsen, 2014, p. 490).
Ethical leadership	Ethical leadership is defined as “the demonstration of normatively appropriate conduct through personal actions and interpersonal relationships, and the promotion of such conduct to followers through two-way communication, reinforcement, and decision-making” (Brown et al., 2005, p. 120).
Abusive supervision	Abusive supervision refers to “subordinates' perceptions of the extent to which supervisors engage in the sustained display of hostile verbal and non-verbal behaviors, excluding physical contact” (Tepper, 2000, p. 178).

To start, we would like to briefly introduce intrinsic motivation. Intrinsic motivation and extrinsic motivation have been widely studied. Extrinsically motivated behaviors are “governed by the prospect of instrumental gain and loss (e.g., incentives), whereas intrinsically motivated behaviors are engaged for their very own sake (e.g., task enjoyment), not being instrumental toward some other outcome” (Cerasoli et al., 2014, p. 1). This definition of intrinsic motivation has been widely accepted in meta-analyses (e.g., Deci et al., 1999; Patall et al., 2008; Cerasoli et al., 2014). Beyond enjoyment-based intrinsic motivation, individuals also are likely to be intrinsically motivated by obligation. That is, obligation-based intrinsic motivation (to meet the morals, values. and ethics dictated by an individual) may exist (Li et al., 2012). This study focus on enjoyment-based intrinsic motivation.

We apply SDT to develop the links between leadership and intrinsic motivation. Drawing on SDT, all human beings have three basic psychological needs, namely, needs for competence, autonomy, and relatedness (Ryan and Deci, 2000b; Gagné and Deci, 2005; Deci and Ryan, 2014). The need for autonomy reflects the need to be the origin of their own behaviors and choices; the need for competence reflects the need to be competent, effective, and masterful; and the need for relatedness reflects the need to feel a sense of meaningful connection with at least some other people (Sheldon and Prentice, 2019). SDT argues that social contexts that satisfy these needs would increase intrinsic motivation (Deci and Ryan, 2000; Ryan and Deci, 2000b).

Based on SDT, leadership would influence basic psychological needs, thus activating intrinsic motivation. First, transformational leaders use intellectual stimulation and individualized consideration to influence their followers. Intellectual stimulation would make their followers innovative, while individual consideration would meet their developmental needs (Bass, 1999). Positive leadership behaviors (i.e., intellectual stimulation and individual consideration) would allow transformational leaders to build a positive relationship with their followers and satisfy their followers' need for relatedness. Second, empowering leaders use multiple behaviors to support their followers' autonomy. For instance, empowering leaders share power with their followers, support subordinates' motivation to work autonomously, and promote subordinates' learning and development in their work roles (Amundsen and Martinsen, 2014). By doing so, empowering leaders are likely to build a positive association with their followers and thereby satisfy the need for relatedness. Third, servant leaders empower their followers. They encourage and facilitate their followers, in identifying and solving problems, and determining when and how to complete work tasks (Liden et al., 2008). As such, servant leaders are likely to build positive relationships with their followers, fulfilling the need for relatedness. Fourth, with a high quality of LMX, employees are likely to have a social exchange relationship including trust, loyalty, and commitment with their leaders (Dulebohn et al., 2012), satisfying the need for relatedness. Finally, ethical leaders show their honesty and trustworthiness to their followers and care for them (Brown and Treviño, 2006), which would help them to build positive relationships with their employees, satisfying their followers' need for the relatedness.

Together, transformational leadership, servant leadership, empowering leadership, LMX, and ethical leadership would satisfy their followers' need for relatedness. According to SDT, when psychological need is satisfied, individuals would be motivated intrinsically (Ryan and Deci, 2000b). Besides, early studies found that transformational leadership (Al Harbi et al., 2019; Mahmood et al., 2019), servant leadership (Kong et al., 2017; Su et al., 2020), empowering leadership (Byun et al., 2016; Ju et al., 2019), LMX (Piccolo and Colquitt, 2006; Xie et al., 2020), and ethical leadership (Yidong and Xinxin, 2012; Potipiroon and Ford, 2017) are positively related to intrinsic motivation.

***Hypothesis 1**:* Transformational leadership (a), servant leadership (b), empowering leadership (c), ethical leadership (d), and LMX (e) will positively relate to intrinsic motivation.

Abusive supervision may harm their relationships with their followers through their abusive behavior. For example, they ridicule their followers and invade their followers' privacy (Tepper, 2000). Drawing on SDT (Deci and Ryan, 2000), abusive supervision would undermine the need for relatedness and thereby decrease their followers' intrinsic motivation. Previous studies found that abusive supervision is negatively linked to intrinsic motivation (Hussain et al., 2020; Onaran and Göncü-Köse, 2022).

***Hypothesis 2**:* Abusive supervision will negatively relate to intrinsic motivation.

### Relative importance of leadership

Although five positive leadership styles may positively relate to intrinsic motivation, it is unclear what kinds of leadership contribute more variance to intrinsic motivation. Based on the need for relatedness, we could not explain which leadership styles might promote more intrinsic motivation. Fortunately, SDT is a very grand theory that includes many mini-theories, based on the organismic integration mini-theory, we try to explain the different impacts of leadership styles on intrinsic motivation. Besides, it seems very hard to compare all leadership together. As such, following early studies (Hoch et al., 2018; Lee et al., 2020a), we use transformational leadership as a benchmark and then compare other leadership with it.

The organismic integration mini-theory argues all motivated behaviors can be located on an underlying autonomy continuum, somewhere between feeling a complete lack of self-determined to feeling completely self-determined (Ryan and Deci, 1989, 2000a). Drawing on SDT, leadership that provides a higher level of autonomy may link to a higher level of intrinsic motivation. First, compared to transformational leadership, empowering leadership may influence motivation that is more autonomous. In particular, transformational leaders may not empower their followers in some situations. For instance, Sharma and Kirkman (2015) argued that leaders may exhibit transformational behavior without actually transferring much control or power to their followers. However, empowering leaders encourages independence and autonomy (Amundsen and Martinsen, 2014). In other words, transformational leadership may undermine autonomy in some situations while empowering leaders may not. Drawing on SDT, the motivation influenced by empowering leadership rather than transformation leadership is more closed to intrinsic motivation. As such, empowering leadership may contribute a larger variance to intrinsic motivation than transformational leadership.

Second, servant leadership may influence a higher degree of autonomous motivation than transformational leadership. Servant leaders' primary focus is on their followers, while transformational leaders primarily focus on organizational objectives (Hoch et al., 2018). As such, servant leaders would consider more interests of their followers and provide more autonomy to their followers than transformational leaders. Besides, the measure of servant leadership includes empowerment (e.g., Liden et al., 2015), while empowerment was removed in the recent measure of transformational leadership (Bass, 1999). Together, compared to transformation leadership, servant leadership may provide more autonomy to their followers, contributing more variance to intrinsic motivation.

Third, ethical leadership may influence a lower degree of autonomous motivation relative to transformational leadership. The ethical leader would punish their followers who violate ethical standards (Brown et al., 2005). Although punishment is necessary in the organization, punishment is a kind of control that might undermine autonomy. Punishment is a kind of behavior in transactional leaders rather than transformational leaders (Bass, 1999). Thus, transformational leadership might undermine less autonomy than ethical leadership, contributing more variance to intrinsic motivation.

Fourth, LMX is a form of relational leadership (Liden and Maslyn, 1998). It does not emphasize empowerment or control. Drawing on SDT, it is quite hard to predict its relative importance to intrinsic motivation relative to transformational leadership. Although SDT could help us to illustrate the relative importance of leadership styles to some extent, similarities of concepts between leadership styles may limit us accurately predicting which leadership styles will contribute a larger part of the variance. For instance, early meta-analyses found large correlations between ethical leadership (*ρ* = 0.70), servant leadership (*ρ* = 0.52), and transformational leadership. As such, we do not propose a hypothesis. Instead, we try to answer the following research question:

***Research Question 1***: Will empowering leadership (a), servant leadership (b), ethical leadership (c), and LMX (d) contribute more variance to intrinsic motivation relative to transformational leadership?

### Moderators of leadership–intrinsic motivation

We choose two cultural dimensions as moderators for two reasons. The first one is that SDT literature (e.g., Chirkov et al., 2003; Church et al., 2013; Deci et al., 2017) focus on individualism and power distance. By researching these two moderators, we could contribute to SDT literature. The second one is that prior meta-analyses about leadership (e.g., Lee et al., 2020a,b) focus on individualism and power distance, suggesting these two moderators are very important in leadership literature.

#### Power distance

Early meta-analyses (Lee et al., 2020a; Li et al., 2021) have found power distance has a moderating effect on leadership effectiveness. Power distance reflects “the extent to which a society accepts the fact that power in institutions and organizations is distributed unequally” (Hofstede, 1980, p. 45). Employees who are in a society with a high-power distance orientation expect direction from their leaders (Javidan et al., 2006). As such, leadership may have a stronger influence on employees in a high power distance country. That is, the association between leadership and intrinsic motivation will be stronger in a country with a higher power distance.

***Hypothesis 3**:* Power distance moderates the association between leadership and intrinsic motivation. In particular, the effect size will be higher when the sample comes from a country with a higher power distance.

#### Individualism

Individualism implies “a loosely knit social framework in which people are supposed to take care of themselves and of their immediate families only” (Hofstede, 1980, p. 45). According to SDT, although all human beings have three basic psychological needs (Deci and Ryan, 2000), individuals vary in internalizing the influence of the environment. In the workplace, the degree of internalization of leaders' influence varies in different cultures. Internalization is relatively low in cultures with high individualism (Chirkov et al., 2003). In other words, when samples from a country with a higher individualism level, the impact of leadership would be weaker.

***Hypothesis 4**:* Individualism moderates the association between leadership and intrinsic motivation. In particular, the effect size will be lower when the sample comes from a country with a higher individualism.

#### Source

When a study uses common source data, namely, independent and dependent variables are collected from a single time point, correlations are likely to inflate due to common method bias (Podsakoff et al., 2003). In contrast, using non-common source data may decrease the influence of common method bias to some extent. Previous meta-analyses (Lee et al., 2020a,b) found that correlations are higher when using common source data.

***Hypothesis 5**:* Source the association between leadership and intrinsic motivation. In particular, the effect size will be higher when the study uses common source data.

#### Journal quality

The peer-review process for a paper published in a higher-quality journal is generally more rigorous. A rigorous peer-review process may influence the quality of data. That is, in a high-level quality journal, data are likely to have higher quality than in a relatively lower-level quality journal. For instance, papers published in the Journal of Applied Psychology (SSCI Q1) are more likely to have a higher quality of data than in a journal that was included in SSCI Q4. We want to know whether the journal quality would influence the correlations of interest.

***Research Question 2***: Does journal quality moderate the links between leadership and intrinsic motivation?

#### Publication year

Since papers published in different years might be influenced by many factors (e.g., economic conditions and research paradigm), we want to know if the publication year moderates the effect sizes.

***Research Question 3***: Does publication year moderate the links between leadership and intrinsic motivation?

## Methods

This research used multiple strategies to identify studies that include relationships between leadership and intrinsic motivation. In particular, searches were conducted in the following databases in March 2022: PsycINFO and Web of Science. We used the following keywords: leadership and intrinsic motivation.

### Inclusion criteria and coding

**Figure 1:**
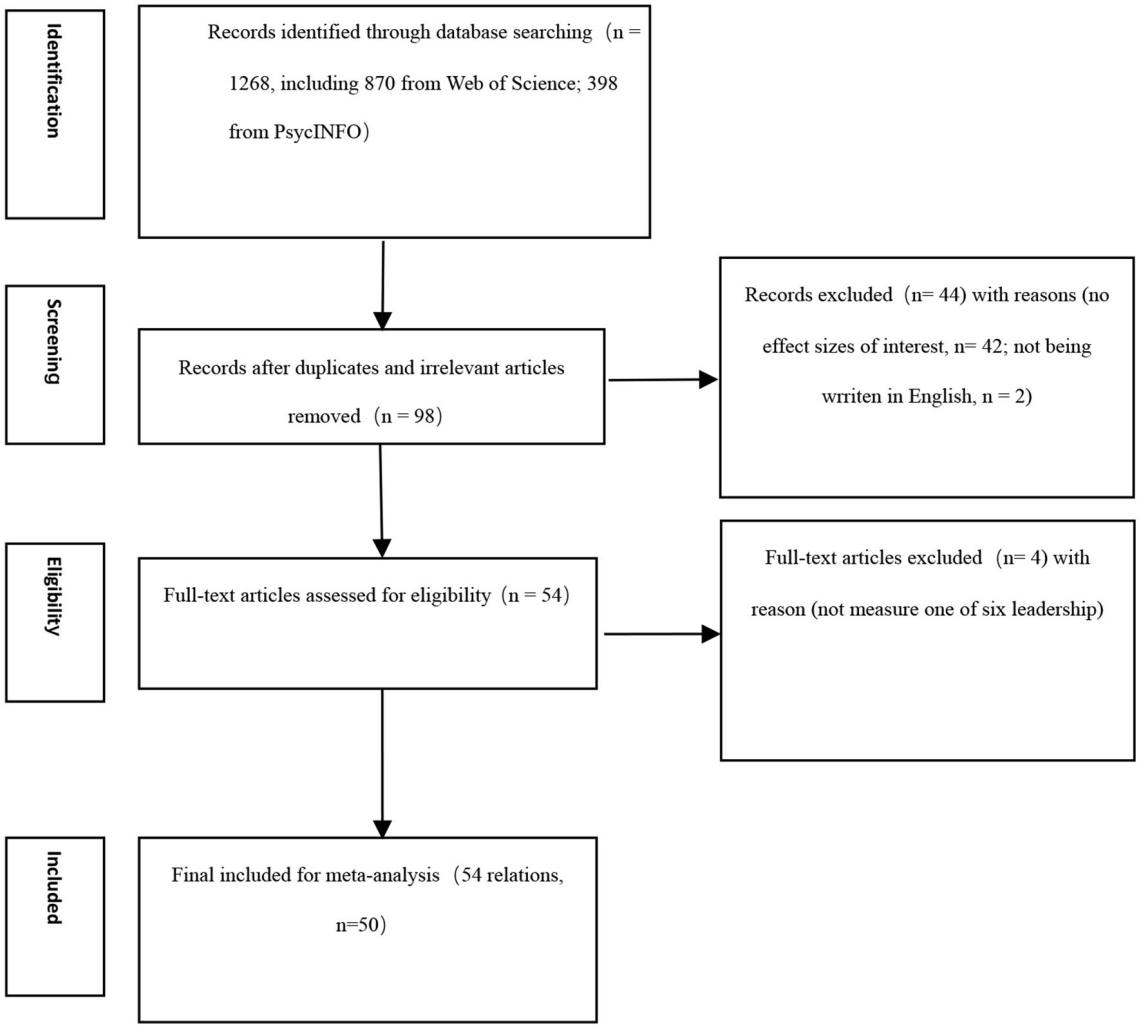
PRISMA flowchart.

We employed several criteria to determine whether to include studies in our analyses. First, the study should be an empirical study that includes correlation(s). For instance, the qualitative review was removed because it does not provide a correlation. Second, studies should be written in English. Third, the sample should come from the workplace. For example, student and athlete samples were removed. Finally, leadership types should be one of the six we mentioned. In the coding process, we noticed some leadership styles have few studies (*k* < 3). As such, these studies were excluded. To illustrate our research process, the PRISMA flowchart is presented in Figure 1.

Two of our authors coded the following information: bibliographic references (authors and publication year), sample description (sample size and country), research design/sampling strategy, effect sizes (correlations), and the reliabilities of all scales. For studies with multiple indicators of a focal construct, we averaged them (Hunter and Schmidt, 2004; Hoch et al., 2018). For example, when one study did not report an overall correlation between leadership and motivation, but only the correlations between dimensions of leadership and motivation, we averaged these correlations to evaluate an overall correlation.

### Analyses

We applied the Hunter-Schmidt method's meta-analysis methodology to correct sampling error and measurement error (Hunter and Schmidt, 2004). In particular, the measurement error was corrected by reliability individually. Cronbach's **α** was employed as the reliability. The details of reliability were shown in Table 2. The sampling error was corrected by the random-effect model. Meta-analysis was performed using the psychmeta package (Dahlke and Wiernik, 2019) in R. The results of meta-analysis were shown in Table 3.

**Table 2:** Cronbach's **α** reliabilities of the current study.

**Variable**	**Number of α**	**Average of α**	**Maximum α**	**Minimum α**	**Sample size weighted average of α**
Intrinsic motivation	54	0.85	0.98	0.68	0.85
Abusive supervision	5	0.89	0.94	0.76	0.89
Empowering leadership	4	0.89	0.96	0.82	0.94
Ethical leadership	6	0.83	0.95	0.71	0.91
LMX	6	0.81	0.96	0.68	0.83
Servant leadership	4	0.9	0.80	0.85	0.88
Transformational leadership	29	0.85	0.68	0.85	0.90

**Table 3:** Bivariate relationships between leadership and intrinsic motivation.

**variable**	***k***	***n***	***r***	**ρ**	**SDρ**	**95% CI**	**80% CR**
Abusive supervision	5	1561	−0.36	−0.42	0.05	[−0.51, −0.32]	[−0.49, −0.34]
Empowering leadership	4	4614	0.40	0.45	0.09	[0.29, 0.60]	[0.30, 0.60]
Ethical leadership	6	1725	0.43	0.49	0.24	[0.23, 0.74]	[0.14, 0.84]
LMX	6	3179	0.30	0.37	0.11	[0.25, 0.50]	[0.21, 0.53]
Servant leadership	4	1315	0.42	0.49	0.19	[0.17, 0.81]	[0.17, 0.80]
Transformational Leadership	29	9852	0.32	0.37	0.2	[0.29, 0.45]	[0.10, 0.63]

k, number of studies; n, total sample size in the meta-analysis; r, uncorrected effect size; ρ, corrected effect size; SDρ, standard deviation of the corrected effect size; CI, confidence interval; CV, credibility interval.

Following the guidance by Tonidandel and LeBreton (2011), we conducted a relative importance analysis. We built a meta-analytic correlation matrix for the relative importance analysis (see Table 4). Then, we applied RWA Web (Tonidandel and LeBreton, 2015) to accomplish this analysis. The results was provided in Table 5.

**Table 4:** Meta-analytic correlation matrix.

**Variable**	**Intrinsic** **motivation**	**Transformational** **leadership**	**Empowering** **leadership**	**Ethical** **sleadership**	**LMX**	**Servant** **leadership**
Intrinsic motivation	1	0.37	0.45	0.49	0.37	0.49
k (*n*)	-	29 (9,852)	4 (4,614)	6 (1,725)	6 (3,179)	4 (1,315)
Transformational leadership	-	1	0.67^b^	0.7^a^	0.71^a^	0.52^a^
k (*n*)	-	-	5 (1,721)	20 (3,717)	20 (4,591)	5 (774)

Unless stated, meta-analytic correlations were calculated by authors.

a Hoch et al., 2018;

b Lee et al., 2018.

**Table 5:** Relative weights analysis.

**Variable**	**Raw relative weights**	**Rescaled Relative weights %**	**R-square**
Transformational leadership	0.07	34.46	0.21
Empowering leadership	0.14	65.54	
Transformational leadership	0.07	28.64	0.24
Ethical Leadership	0.17	71.36	
Transformational leadership	0.08	50.24	0.16
LMX	0.08	49.76	
Transformational leadership	0.08	30.02	0.26
Servant leadership	0.18	69.98	

We employed meta-regression to detect the potential moderating effects of power distance, individualism, source, journal quality, and publication year. The index of power distance and individualism was extracted from Hofstede's website (www.geerthofstede.com). Source was coded as a dummy variable. In particular, “common source” was coded as “0,” while “non-common source” was coded as “1.” Journal quality was coded according to the journal rank. For instance, if one paper is published in SSCI Q4, it would be coded as 4; if one paper is published in SSCI Q1, it would be coded as 1. The regression was accomplished using metafor (Viechtbauer, 2010) package in R. In particular, we employed a random-effect model and regarded Restricted Maximum Likelihood (REML) method as an estimator to conduct our meta-regression. The results were presented in Table 6.

**Table 6:** Moderation analyses.

**Variable**	**Moderator**	**Estimate**	***Z***	***p***	**Moderator effect present?**
Abusive supervision	Year	0	−0.06	0.925	No
	Source	0.13	1.45	0.148	No
	Quality of journal	−0.05	−2.03	0.042	Yes, the lower the quality of the journal, the larger the magnitude of correlation
	Power distance	0	0.95	0.343	No
	Individulism	0	0.99	0.324	No
Empowering leadership	Year	0.02	0.64	0.52	No
	Source	0.08	0.27	0.788	No
	Quality of journal	0.15	4.82	0	Yes, the lower the quality of the journal, the larger the magnitude of correlation
	Power distance	0	−0.19	0.851	No
	Individulism	0	−0.74	0.458	No
Ethical leadership	Year	0.07	1.66	0.097	Yes, the larger the year, the larger the correlation
	Source	0.03	0.13	0.9	No
	Quality of journal	0	0.04	0.969	No
	Power distance	0	0.52	0.606	No
	Individulism	0	−0.75	0.454	No
LMX	Year	0.01	0.94	0.35	No
	Source	-	-	-	-
	Quality of journal	−0.04	−0.62	0.539	No
	Power distance	0	−0.12	0.907	No
	Individulism	0	0.04	0.967	No
Servant leadership	Year	0.13	6.51	0	Yes, the larger the year, the larger the correlation
	Source	0.06	0.19	0.853	No
	Quality of journal	−0.05	−0.31	0.753	No
	Power distance	−0.01	−1.66	0.097	Yes, the larger the power distance, the smaller the correlation
	Individulism	0	0.06	0.952	No
Transformational leadership	Year	0.01	1.44	0.149	No
	Source	−0.08	−0.48	0.633	No
	Quality of journal	0.06	1.45	0.142	No
	Power distance	0	0.92	0.357	No
	Individulism	0	0.04	0.967	No

Finally, publication bias occurs because statistically significant results are published more frequently than studies without significant results (Rothstein et al., 2005). To ensure the robustness of the current study, we applied the trim-and-fill method (Duval and Tweedie, 2000) and Eggs' regression to detect publication bias (see Table 7).

**Table 7:** Publication bias analysis.

**Variable**	**Trim-and-Fill**				**Egg's regression**			
	**Observed k**	**Unadj. r+**	**Imputed k**	**Adj. r+**	**Change**	**t**	**df**	**p**
Abusive supervision	5	−0.37	1	−0.36	0.01	0.78	3	0.495
Empowering leadership	4	0.38	0	0.38	0	−0.5	2	0.669
Ethical leadership	6	0.41	1	0.44	0.03	−2.02	4	0.114
LMX	6	0.38	0	0.38	0	2.29	4	0.084
Servant leadership	4	0.42	0	0.42	0	−1.23	2	0.345
Transformational leadership	29	0.36	0	0.36	0	1.7	27	0.101

Observed k, number of aggregated effect sizes included in analyses; Unadj. r+, unadjusted effect size estimate; imputed k, number of additional effect sizes added by trim-and-fill analyses; Adj. r+, adjusted effect size estimate (i.e., including imputed studies).

## Results

As shown in Table 3, we find that abusive supervision (*ρ* = −0.42, 95%CI = [−0.51, −0.32]) is negatively related to intrinsic motivation. Transformational leadership (*ρ* = 0.37, 95%CI = [0.29, 0.45]), ethical leadership (*ρ* = 0.49, 95%CI = [0.23, 0.74]), servant leadership (*ρ* = 0.49, 95%CI = [0.17, 0.81]), empowering leadership (*ρ* = 0.45, 95%CI = [0.29, 0.60]), and LMX (*ρ* = 0.37, 95%CI = [0.25, 0.50]) are positively related to intrinsic motivation. Thus, H1 (a), H1 (b), H1 (c), H1 (d), H1 (e), and H2 are accepted.

As presented in Table 5, empowering leadership (65.54%) played a more important role in explaining intrinsic motivation than transformational leadership (34.46%). Similarly, ethical leadership (71.36%) explained a larger portion of the variance than transformational leadership (28.64%). LMX (49.76%) and transformational leadership (50.24%) played a similar role in explaining intrinsic motivation. Servant leadership (69.98%) played a more important role than transformational leadership (30.02%). Together, RQ1 was answered.

As illustrated in Table 4, we did not find evidence that supports the moderating effects of individualism and source. Regarding publication year, we found that the links between ethical (servant) leadership and intrinsic motivation were larger when the publication year was larger. Interestingly, for abusive supervision and empowering leadership, the correlation became larger as the journal quality became lower. These results answer RQ 2. In terms of power distance, we noticed that the correlation between servant leadership and intrinsic motivation became smaller when power distance became larger. These results answer RQ 3. Therefore, H4 and H5 were rejected, while H3 was accepted partly.

Finally, as depicted in Table 7, the overall publication is not serious. First, drawing on Egg's regression method, among six leadership styles, all the *p*-value is bigger than 0.050, suggesting publication bias is not series. Second, the Trim-and-Fill method helps to fill asymmetric effect sizes and provides an adjusted overall effect size. In terms of empowering leadership, LMX, servant leadership, and transformational leadership, no asymmetric effect sizes were found. Regarding abusive supervision, effect size only changes by 0.01 after adjusting asymmetric effect sizes. For ethical leadership, effect size only changes by 0.03 after adjusting asymmetric effect sizes. Together, we did not find large changes after using the Trim-and-Fill method, confirming the robustness of the current meta-analysis.

## Discussion

Given the importance of intrinsic motivation in work, it is critical to understand the leadership–intrinsic motivation association. This study aimed to contribute to the leadership and intrinsic motivation literature by estimating the true population correlations between leadership styles and intrinsic motivation, comparing the relative importance of leadership to intrinsic motivation, and detecting the potential moderators of the relationship between leadership and intrinsic motivation. We discuss our findings in relation to our three key aims.

### True population correlations

Cohen (2013) provided a standard to understand the magnitude of correlations. That is, small effect sizes are correlations of 0.10, moderate are 0.30, and large are 0.50. We applied this standard to discuss the magnitude of effect sizes. We found that abusive supervision (*ρ* = −0.42) is moderately and negatively related to intrinsic motivation. Early meta-analyses (Mackey et al., 2015; Zhang and Liao, 2015) has found abusive supervision is positively related to a series of bad consequence such as counterproductive work behavior, emotional exhaustion, and so on. Our study enriches the understanding of the negative outcomes of abusive supervision, that is, abusive supervision is negatively associated with intrinsic motivation. Besides, it is worth mentioning that this correlation is large, indicating managers could not ignore the bad impact of abusive supervision on intrinsic motivation.

Transformational leadership (*ρ* = 0.37) and LMX (*ρ* = 0.37) are moderately and positively related to intrinsic motivation. These findings highlight the importance of these two leadership styles in organizations. Transformational leadership and LMX have been researched for more than 40 years. Our meta-analysis first quantitatively and accurately estimated their links with intrinsic motivation, contributing to transformational leadership and LMX literature. In the relationship between transformational leadership and intrinsic motivation, we noticed that one study (Li et al., 2012) measured intrinsic motivation using the obligation-based measure. We conducted a sensitivity analysis (leave-one-out analysis) to detect whether the measure of intrinsic motivation would influence the robustness of the results. We found that **k** changed from 29 to 28 and ***n*** changed from 9,852 to 9,734 after removing this study. However, ***r*** and ***ρ*** did not change, suggesting the robustness of the result. That is to say, the measure of intrinsic motivation did not influence the robustness of the current study. This result should be explained carefully because (a) these two kinds of definitions of intrinsic motivation are different to some extent and (b) only one study may not make us capture such influence when applying sensitivity analysis.

Ethical leadership (*ρ* = 0.49), servant leadership (*ρ* = 0.49), and empowering leadership (*ρ* = 0.45) is positively and largely related to intrinsic motivation. Compared to transformational leadership and LMX, ethical leadership, servant leadership, and empowering leadership are three emerging forms of positive leadership and have been studied recently. The twenty-first century is the era of the knowledge economy, more and more jobs need intrinsic motivation. Thus, organizations need to provide employees with more autonomy. Using three positive leadership could be a good choice to provide autonomy to employees.

### Relative importance

We found that empowering and servant leadership explain a larger variance in intrinsic motivation than transformational leadership. According to SDT (Deci and Ryan, 2010; Deci et al., 2017), when individuals are motivated intrinsically, they are likely to be creative and innovative. Lee et al. (2020a) also found similar findings that empowering and servant leadership explain a larger variance in creativity than transformational leadership. Together, our findings provide solid evidence that servant and empowering leadership is important for individuals' intrinsic motivation.

LMX and transformational leadership had a similar role in explaining intrinsic motivation. Interestingly, Lee et al. (2020a) finds LMX (*ρ* = 0.34) and transformational leadership (*ρ* = 0.31) have similar correlations with creativity. The theories and measures of these two leadership styles are quite different. Perhaps they both affect the needs for relatedness, causing them to have similar effects on intrinsic motivation and creativity.

Transformational leadership explained less variance in intrinsic motivation than ethical leadership, which is out of our expectation. In our hypothesis, we believed that ethical leadership may influence less autonomy than transformational leadership, causing ethical leadership to contribute less variance than transformational leadership. Lee et al. (2020a) found ethical leadership explains a larger variance than transformational leadership in creativity. Ethical leadership is a kind of moral leadership. Why a moral leadership would contribute to more variance in intrinsic motivation? SDT may provide an explanation. SDT argues that three psychological needs independently influence intrinsic motivation (Deci et al., 2017). This argument has been confirmed by meta-analytic evidence (Slemp et al., 2018). Transformational leaders may not be ethical and abusive to their followers in some situations (Hoch et al., 2018), which may harm the need for relatedness and thereby decrease intrinsic motivation. As such, ethical leadership may influence a larger need for relatedness than transformational leadership. Together, more theoretical explanations and evidence are called to explain the links between ethical (transformational) leadership and intrinsic motivation.

### Moderators

#### Power distance

In line with early studies (Lee et al., 2020a,b; Lyubykh et al., 2022), power distance has been found to moderate leadership effectiveness. As such, leadership should be contingent according to culture. That is, there is no single type of leadership that works in all cultural situations. This point is especially important in multinational companies as the same leadership may have different effects in different cultures.

#### Individualism

We did not find evidence that individualism has a moderating effect. This finding may suggest intrinsic motivation is a more universal concept. Intrinsic motivation is based on the enjoyment of the process rather than the consequence (Deci and Ryan, 2010). However, individualism is more likely to focus on the consequence immediately (Hofstede, 1980). As such, whether in a low or high individualism country, individuals may be motivated intrinsically equally due to the enjoyment of the process rather than the consequence, and thereby not be influenced by individualism.

#### Source

Results did not support that source has a moderating effect. Although studies using common source data would suffer from common method bias (Podsakoff et al., 2003), these effects in the current study are not series. Nonetheless, we still recommend using time-lagged research designs to reduce the effect of common method bias.

#### Publication year

We noticed that publication year had a moderating effect on some leadership. However, these findings should be explained carefully. Publication year may be associated with a lot of factors. For example, publication year may be linked to economy and management level that may influence leadership and motivation. Besides, publication year may be related to research quality as research quality may increase as time goes by. Together, the moderating effect of the publication year should be understood cautiously.

#### Journal quality

We noticed that the correlation became larger as the journal quality became lower. This finding is in line with our research experience. That is, the data quality would be higher in a journal with higher quality. When data quality is low, they tended to exhibit higher correlations due to an unrigorous research design. Unfortunately, few meta-analyses researched the modering effect of journal quality. We look forward to more meta-analyses focusing on this moderator variable

### Practice implications

The current study also contributes to practice. Drawing on our findings, some management suggestions should be mentioned. First, managers should avoid using abusive supervision in the workplace. In the era of the knowledge economy, intrinsic motivation is very important to the employees' performance quality. However, abusive supervision would undermine intrinsic motivation deeply as the current study finds a strong and negative association between abusive supervision. Second, the organization should provide leadership training programs to managers. In particular, drawing on our findings, ethical, servant, and empowering leadership positively relate to intrinsic motivation. However, many managers are still lacking systematic leadership training. They just manage their followers according to their experience. The human resource department should provide these leadership training programs to managers. Finally, leaders should provide an antonomy support climate to their followers, increasing their followers' intrinsic motivation.

### Limitations and future research directions

Two limitations should be mentioned. First, since the effect sizes used in this study are correlation coefficients, we could not make a valid causal inference. Although it is unlikely that reverse causality exists, for example, employee motivation influencing leadership, there may be a common factor that affects both leadership and employee motivation at the same time. For instance, organizational culture may influence both leadership and employee motivation at the same time. Future studies should use more experiment research designs to make accurate causality between leadership and intrinsic motivation.

Second, multicollinearity may harm the robustness of the current study. One positive leadership is usually highly correlated with other positive leadership, which in turn, may cause multicollinearity. For example, Hoch et al. (2018) find ethical (*ρ* = 0.70) and servant (*ρ* = 0.52) leadership are largely related to transformation leadership. Carlson and Herdman (2010) suggested that convergent validity is well when *r* is bigger than 0.7. In other words, measures of multiple leadership styles have well-convergent validity and they may reflect the same construct to some extent. At the same time, with the influence of multicollinearity, the links between leadership and intrinsic motivation might be biased. For instance, B leadership rather than A leadership is related to intrinsic motivation theoretically. However, due to the high correlation between A and B leadership, A leadership is found to be related to intrinsic motivation. As such, the link between A leadership and intrinsic motivation could be biased. Future studies should use more effective measures to decrease multicollinearity and make a clearer distinction between leadership and its influence on intrinsic motivation.

## Conclusion

Leadership is important for the followers' intrinsic motivation. Although fruitful evidence has been accumulated, some unsolved issues still exist. To address these, the current study provides the first analysis between leadership and intrinsic motivation. Overall, positive leadership (e.g., transformational leadership and servant leadership) positively relate to intrinsic motivation, while abusive supervision negatively relates to intrinsic motivation. Empowering, ethical, and servant leadership explain a larger variance in intrinsic motivation than transformational leadership. Power distance, publication year, and journal quality moderates the association between leadership and intrinsic motivation. Our research enriches our understanding of the relationship between leadership and intrinsic motivation. We also provide some practice suggestions for managers drawing on our findings.

## Data availability statement

The original contributions presented in the study are included in the article/Supplementary materials, further inquiries can be directed to the corresponding author/s.

## Author contributions

HX: idea. HX and YLuan: introduction. HX and YLuo: hypotheses. YLuo, YLuan, and NW: method. NW: result. All authors contributed to the article and approved the submitted version.

## Conflict of interest

The authors declare that the research was conducted in the absence of any commercial or financial relationships that could be construed as a potential conflict of interest.

## Publisher's note

All claims expressed in this article are solely those of the authors and do not necessarily represent those of their affiliated organizations, or those of the publisher, the editors and the reviewers. Any product that may be evaluated in this article, or claim that may be made by its manufacturer, is not guaranteed or endorsed by the publisher.
