# Supplementary material for: A meta-analysis of leadership and intrinsic motivation: Examining relative importance and moderators
Source: Front Psychol. 2022 Aug 12;13:941161. doi: 10.3389/fpsyg.2022.941161 (PMC9413051; doi:10.3389/fpsyg.2022.941161)
Supplement: Supplementary file 1 [file Table_1.DOCX]

Table S1 coding information

| **NO.** | **name** | **year** | **n** | **r** | **α1** | **leadership type** | **α2** | **source** | **Country/Area** |
| --- | --- | --- | --- | --- | --- | --- | --- | --- | --- |
| 1 | Weilin Su | 2020 | 381 | 0.452 | 0.843 | Servant leadership | 0.804 | 1 | China |
| 2 | Shenghao Guo | 2021 | 346 | 0.376 | 0.961 | Ethical leadership | 0.952 | 1 | China |
| 3 | C. Christopher Lee | 2022 | 489 | 0.7 | 0.94 | Transformational Leadership | 0.93 | 0 | USA |
| 4 | Akriti Chaubey | 2019 | 251 | 0.589 | 0.887 | Transformational Leadership | 0.884 | 0 | India |
| 5 | Le Minh-Duc | 2019 | 279 | 0.726 | 0.93 | Transformational leadership | 0.89 | 0 | Vietnam |
| 6 | Brigitte Kroon | 2017 | 382 | 0.29 | 0.92 | Transformational Leadership | 0.93 | 0 | Netherlands |
| 7 | Giang Hoang | 2022 | 415 | 0.729 | 0.92 | Ethical leadership | 0.75 | 0 | Vietnam |
| 8 | Monowar Mahmood | 2019 | 400 | 0.569 | 0.933 | Transformational Leadership | 0.982 | 0 | Bangladesh |
| 9 | Thi Phuong Linh Nguyen | 2022 | 420 | 0.032 | 0.845 | Transformational Leadership | 0.758 | 0 | Vietnam |
| 10 | Adie Irwan Kusumah | 2021 | 531 | 0.24 | 0.928 | Transformational leadership | 0.923 | 0 | Yogyakarta |
| 11 | Transformation leadership | 2019 | 503 | 0.304 | 0.93 | Transformational Leadership | 0.74 | 0 | Saudi Arabia |
| 12 | Zhen Shao | 2016 | 117 | 0.33 | 0.96 | Transformational Leadership | 0.95 | 0 | China |
| 13 | Auks_e Endriulaitien | 2020 | 250 | 0.23 | 0.95 | Transformational leadership | 0.81 | 0 | Lithuania |
| 14 | Annam Bibi | 2018 | 337 | 0.34 | 0.72 | LMX | 0.84 | 0 | Pakistan |
| 15 | L. M. Schopman | 2016 | 171 | 0.62 | 0.92 | LMX | 0.96 | 0 | Dutch |
| 15 | L. M. Schopman | 2016 | 171 | 0.71 | 0.93 | Transformational leadership | 0.96 | 0 | Dutch |
| 16 | Kanwal Hussain | 2020 | 225 | -0.437 | 0.76 | Abusive supervision | 0.9 | 0 | Pakistan |
| 17 | SHUNG JAE SHIN | 2003 | 290 | 0.19 | 0.93 | Transformational leadership | 0.84 | 0 | Korean |
| 18 | Hongping Zhang | 2012 | 235 | -0.32 | 0.94 | Abusive supervision | 0.86 | 0 | China |
| 19 | Frank D. Belschak | 2013 | 141 | 0.47 | 0.92 | Transformational Leadership | 0.9 | 0 | Netherlands |
| 20 | Rui Li | 2019 | 136 | -0.25 | 0.92 | Abusive supervision | 0.83 | 1 | China |
| 21 | Ulrich Thy Jensen | 2017 | 1481 | 0.162 | 0.887 | Transformational Leadership | 0.842 | 0 | Denmark |
| 22 | Laura Thomas | 2020 | 292 | 0.245 | 0.93 | Transformational Leadership | 0.88 | 0 | Belgium |
| 23 | Stacey M. Conchie | 2013 | 251 | 0.31 | 0.93 | Transformational Leadership | 0.75 | 0 | U.K |
| 23 | Stacey M. Conchie | 2013 | 220 | 0.35 | 0.92 | Transformational Leadership | 0.77 | 0 | U.K |
| 24 | RONALD F. PICCOLO | 2006 | 202 | 0.4 | 0.93 | LMX | 0.68 | 0 | USA |
| 24 | RONALD F. PICCOLO | 2006 | 202 | 0.33 | 0.96 | Transformational Leadership | 0.68 | 0 | USA |
| 25 | Naveed Ahmad Faraz | 2021 | 323 | 0.584 | 0.89 | Servant leadership | 0.86 | 0 | Pakistan |
| 26 | Bilal Bin Saeed | 2019 | 347 | 0.34 | 0.79 | Transformational Leadership | 0.82 | 0 | China |
| 26 | Bilal Bin Saeed | 2019 | 393 | 0.27 | 0.83 | Transformational Leadership | 0.92 | 1 | Pakistan |
| 27 | Jie Feng | 2018 | 258 | 0.48 | 0.91 | Ethical leadership | 0.93 | 1 | China |
| 28 | Dong Ju | 2019 | 3717 | 0.426 | 0.94 | Empowering leadership | 0.89 | 1 | China |
| 29 | Zhiyu Xie | 2020 | 386 | 0.39 | 0.85 | LMX | 0.86 | 0 | China |
| 30 | Tu Yidong | 2020 | 302 | 0.243 | 0.823 | Ethical leadership | 0.716 | 0 | Lithuania |
| 31 | PAMELA TIERNEY | 1999 | 159 | 0.26 | 0.91 | LMX | 0.74 | 0 | USA |
| 32 | Mohsin Shafi | 2020 | 164 | 0.308 | 0.886 | Transformational Leadership | 0.949 | 0 | Pakistan |
| 33 | Yan Li | 2012 | 118 | 0.381 | 0.89 | Transformational Leadership | 0.88 | 0 | mixed |
| 34 | Yeunjae Lee | 2018 | 306 | 0.25 | 0.89 | Empowering leadership | 0.88 | 0 | Italy |
| 35 | Mathilde Brière | 2020 | 344 | 0.45 | 0.94 | Servant leadership | 0.9 | 0 | France |
| 36 | MING KONG | 2017 | 267 | 0.13 | 0.834 | Servant leadership | 0.824 | 0 | China |
| 37 | Jeremy Chua | 2019 | 155 | 0.319 | 0.97 | Transformational Leadership | 0.83 | 1 | Australia |
| 38 | C. Logan Chullen | 2010 | 1924 | 0.24 | 0.82 | LMX | 0.8 | 0 | USA |
| 39 | Mariam Masood | 2017 | 587 | 0.25 | 0.86 | Transformational leadership | 0.84 | 0 | Pakistan |
| 40 | Beate J. Løvaas | 2020 | 252 | 0.2 | 0.77 | Transformational leadership | 0.87 | 0 | Norway |
| 41 | Sami Okan Onaran | 2022 | 425 | -0.33 | 0.93 | Abusive supervision | 0.78 | 0 | Turkey |
| 42 | Azis E. | 2019 | 300 | 0.525 | 0.806 | Transformational leadership | 0.719 | 0 | Indonesia |
| 43 | Hussain Tariq | 2016 | 540 | -0.39 | 0.88 | Abusive supervision | 0.82 | 0 | China |
| 44 | XIAOMENG ZHANG | 2010 | 367 | 0.2 | 0.92 | Empowering leadership | 0.82 | 0 | China |
| 45 | Kimberly S. Jaussi | 2003 | 322 | -0.04 | 0.95 | Transformational Leadership | 0.79 | 0 | USA |
| 46 | Hira Khan | 2020 | 308 | 0.29 | 0.84 | Transformational Leadership | 0.89 | 0 | Pakistan |
| 47 | GUKDO BYUN | 2016 | 224 | 0.59 | 0.97 | Empowering leadership | 0.96 | 0 | South Korea |
| 48 | Li Yi | 2019 | 236 | 0.471 | 0.89 | Transformational Leadership | 0.766 | 0 | China |
| 49 | Wisanupong Potipiroon | 2017 | 196 | 0.26 | 0.98 | Ethical leadership | 0.92 | 0 | Thailand |
| 50 | Yidong Tu | 2016 | 208 | 0.249 | 0.899 | Ethical leadership | 0.705 | 0 | China |

Note. We only show the first author. In relation to source, the common source is coded as “0”, while the non-common source is coded as “1”.

Reference

Al Harbi, J. A., Alarifi, S., & Mosbah, A. (2019). Transformation leadership and creativity. *Personnel Review, 48*(5), 1082-1099. doi:10.1108/pr-11-2017-0354

Azis, E., Prasetio, A. P., Gustyana, T. T., Putril, S. F., & Rakhmawati, D. (2019). The Mediation of Intrinsic Motivation and Affective Commitment in the Relationship of Transformational Leadership and Employee Engagement in Technology-Based Companies. *Polish Journal of Management Studies, 20*(1), 54-63. doi:10.17512/pjms.2019.20.1.05

Belschak, F. D., Den Hartog, D. N., & Kalshoven, K. (2013). Leading Machiavellians. *Journal of Management, 41*(7), 1934-1956. doi:10.1177/0149206313484513

Bibi, A., & Afsar, B. (2018). Leader-member exchange and innovative work behavior: The role of intrinsic motivation, psychological empowerment, and creative process engagement. *Perspectives of Innovations, Economics and Business, 18*(1), 25-43. doi:10.15208/pieb.2018.3

Bin Saeed, B., Afsar, B., Shahjehan, A., & Imad Shah, S. (2019). Does transformational leadership foster innovative work behavior? The roles of psychological empowerment, intrinsic motivation, and creative process engagement. *Economic Research-Ekonomska Istraživanja, 32*(1), 254-281. doi:10.1080/1331677x.2018.1556108

Brière, M., Le Roy, J., & Meier, O. (2021). Linking servant leadership to positive deviant behavior: The mediating role of self‐determination theory. *Journal of Applied Social Psychology, 51*(2), 65-78.

Byun, G., Dai, Y., Lee, S., & Kang, S.-W. (2016). When Does Empowering Leadership Enhance Employee Creativity? A Three-way Interaction Test. *Social Behavior and Personality: an international journal, 44*(9), 1555-1564. doi:10.2224/sbp.2016.44.9.1555

Chaubey, A., & Sahoo, C. K. (2019). Enhancing organizational innovation in Indian automobile industry. *International Journal of Innovation Science, 11*(1), 82-101. doi:10.1108/ijis-02-2018-0022

Chua, J., & Ayoko, O. B. (2019). Employees’ self-determined motivation, transformational leadership and work engagement. *Journal of Management & Organization, 27*(3), 523-543. doi:10.1017/jmo.2018.74

Chullen, C. L., Dunford, B. B., Angermeier, I., Boss, R. W., & Boss, A. D. (2010). Minimizing deviant behavior in healthcare organizations: The effects of supportive leadership and job design. *Journal of Healthcare Management, 55*(6), 381-397.

Conchie, S. M. (2013). Transformational leadership, intrinsic motivation, and trust: a moderated-mediated model of workplace safety. *J Occup Health Psychol, 18*(2), 198-210. doi:10.1037/a0031805

Endriulaitiene, A., & Morkeviciute, M. (2020). The Unintended Effect of Perceived Transformational Leadership Style on Workaholism: The Mediating Role of Work Motivation. *J Psychol, 154*(6), 446-465. doi:10.1080/00223980.2020.1776203

Faraz, N. A., Ahmed, F., Ying, M., & Mehmood, S. A. (2021). The interplay of green servant leadership, self‐efficacy, and intrinsic motivation in predicting employees’ pro‐environmental behavior. *Corporate Social Responsibility and Environmental Management, 28*(4), 1171-1184. doi:10.1002/csr.2115

Feng, J., Zhang, Y., Liu, X., Zhang, L., & Han, X. (2016). Just the Right Amount of Ethics Inspires Creativity: A Cross-Level Investigation of Ethical Leadership, Intrinsic Motivation, and Employee Creativity. *Journal of Business Ethics, 153*(3), 645-658. doi:10.1007/s10551-016-3297-1

Guo, S., & Hu, Q. (2021). Be zhongyong and be ethical: dual leadership in promoting employees’ thriving at work. *Chinese Management Studies*. doi:10.1108/cms-02-2021-0053

Hoang, G., Luu, T. T., Du, T., & Nguyen, T. T. (2022). Can both entrepreneurial and ethical leadership shape employees’ service innovative behavior? *Journal of Services Marketing*. doi:10.1108/jsm-07-2021-0276

Hussain, K., Abbas, Z., Gulzar, S., Jibril, A. B., Hussain, A., & Foroudi, P. (2020). Examining the impact of abusive supervision on employees’ psychological wellbeing and turnover intention: The mediating role of intrinsic motivation. *Cogent Business & Management, 7*(1). doi:10.1080/23311975.2020.1818998

Jaussi, K. S., & Dionne, S. D. (2003). Leading for creativity: The role of unconventional leader behavior. *The Leadership Quarterly, 14*(4-5), 475-498. doi:10.1016/s1048-9843(03)00048-1

Jensen, U. T., & Bro, L. L. (2017). How Transformational Leadership Supports Intrinsic Motivation and Public Service Motivation: The Mediating Role of Basic Need Satisfaction. *The American Review of Public Administration, 48*(6), 535-549. doi:10.1177/0275074017699470

Ju, D., Ma, L., Ren, R., & Zhang, Y. (2019). Empowered to Break the Silence: Applying Self-Determination Theory to Employee Silence. *Front Psychol, 10*, 485. doi:10.3389/fpsyg.2019.00485

Khan, H., Rehmat, M., Butt, T. H., Farooqi, S., & Asim, J. (2020). Impact of transformational leadership on work performance, burnout and social loafing: a mediation model. *Future Business Journal, 6*(1). doi:10.1186/s43093-020-00043-8

Kong, M., Xu, H., Zhou, A., & Yuan, Y. (2017). Implicit followership theory to employee creativity: The roles of leader–member exchange, self-efficacy and intrinsic motivation. *Journal of Management & Organization, 25*(1), 81-95. doi:10.1017/jmo.2017.18

Kroon, B., van Woerkom, M., & Menting, C. (2017). Mindfulness as substitute for transformational leadership. *Journal of Managerial Psychology, 32*(4), 284-297. doi:10.1108/jmp-07-2016-0223

Kusumah, A. I., Haryadi, Indrayanto, A., & Setiawan, I. (2021). A mediating and moderating role on employee performance appraisal. *Management Research Review, 44*(12), 1639-1659. doi:10.1108/mrr-11-2020-0701

Lee, C. C., Lim, H. S., Seo, D., & Kwak, D.-H. A. (2022). Examining employee retention and motivation: the moderating effect of employee generation. *Evidence-based HRM: a Global Forum for Empirical Scholarship*. doi:10.1108/ebhrm-05-2021-0101

Lee, Y., Mazzei, A., & Kim, J.-N. (2018). Looking for motivational routes for employee-generated innovation: Employees' scouting behavior. *Journal of Business Research, 91*, 286-294. doi:10.1016/j.jbusres.2018.06.022

Li, R., Chen, Z., Zhang, H., & Luo, J. (2019). How Do Authoritarian Leadership and Abusive Supervision Jointly Thwart Follower Proactivity? A Social Control Perspective. *Journal of Management, 47*(4), 930-956. doi:10.1177/0149206319878261

Li, Y., Tan, C.-H., & Teo, H.-H. (2012). Leadership characteristics and developers’ motivation in open source software development. *Information & Management, 49*(5), 257-267. doi:10.1016/j.im.2012.05.005

Løvaas, B. J., Jungert, T., Van den Broeck, A., & Haug, H. (2020). Does managers' motivation matter? Exploring the associations between motivation, transformational leadership, and innovation in a religious organization. *Nonprofit Management and Leadership, 30*(4), 569-589. doi:10.1002/nml.21405

Mahmood, M., Uddin, M. A., & Fan, L. (2019). The influence of transformational leadership on employees’ creative process engagement. *Management Decision, 57*(3), 741-764. doi:10.1108/md-07-2017-0707

Masood, M., & Afsar, B. (2017). Transformational leadership and innovative work behavior among nursing staff. *Nurs Inq, 24*(4). doi:10.1111/nin.12188

Minh-Duc, L., & Huu-Lam, N. (2019). Transformational leadership, customer citizenship behavior, employee intrinsic motivation, and employee creativity. *Journal of Asian Business and Economic Studies, 26*(2), 286-300. doi:10.1108/jabes-10-2018-0070

Nguyen, T. P. L., Nguyen, T. T., Duong, C. D., & Doan, X. H. (2022). The effects of transformational leadership on employee creativity in Vietnam telecommunications enterprises. *Management Decision, 60*(3), 837-857. doi:10.1108/md-07-2020-0882

Onaran, S. O., & Göncü-Köse, A. (2022). Mediating processes in the relationships of abusive supervision with instigated incivility, CWBs, OCBs, and multidimensional work motivation. *Current Psychology*. doi:10.1007/s12144-022-03128-5

Piccolo, R. F., & Colquitt, J. A. (2006). Transformational leadership and job behaviors: The mediating role of core job characteristics. *Academy of management Journal, 49*(2), 327-340.

Potipiroon, W., & Ford, M. T. (2017). Does Public Service Motivation Always Lead to Organizational Commitment? Examining the Moderating Roles of Intrinsic Motivation and Ethical Leadership. *Public Personnel Management, 46*(3), 211-238. doi:10.1177/0091026017717241

Schopman, L. M., Kalshoven, K., & Boon, C. (2015). When health care workers perceive high-commitment HRM will they be motivated to continue working in health care? It may depend on their supervisor and intrinsic motivation. *The International Journal of Human Resource Management, 28*(4), 657-677. doi:10.1080/09585192.2015.1109534

Shafi, M., Zoya, Lei, Z., Song, X., & Sarker, M. N. I. (2020). The effects of transformational leadership on employee creativity: Moderating role of intrinsic motivation. *Asia Pacific Management Review, 25*(3), 166-176. doi:10.1016/j.apmrv.2019.12.002

Shao, Z., Feng, Y., & Wang, T. (2016). Charismatic leadership and tacit knowledge sharing in the context of enterprise systems learning: the mediating effect of psychological safety climate and intrinsic motivation. *Behaviour & Information Technology, 36*(2), 194-208. doi:10.1080/0144929x.2016.1221461

Shin, S. J., & Zhou, J. (2003). Transformational leadership, conservation, and creativity: Evidence from Korea. *Academy of management Journal, 46*(6), 703-714.

Su, W., Lyu, B., Chen, H., & Zhang, Y. (2020). How does servant leadership influence employees' service innovative behavior? The roles of intrinsic motivation and identification with the leader. *Baltic Journal of Management, 15*(4), 571-586. doi:10.1108/bjm-09-2019-0335

Tariq, H., & Ding, D. (2018). Why am I still doing this job? The examination of family motivation on employees’ work behaviors under abusive supervision. *Personnel Review, 47*(2), 378-402. doi:10.1108/pr-07-2016-0162

Thomas, L., Tuytens, M., Devos, G., Kelchtermans, G., & Vanderlinde, R. (2018). Transformational school leadership as a key factor for teachers’ job attitudes during their first year in the profession. *Educational Management Administration & Leadership, 48*(1), 106-132. doi:10.1177/1741143218781064

Tierney, P., Farmer, S. M., & Graen, G. B. (1999). An examination of leadership and employee creativity: The relevance of traits and relationships. *Personnel psychology, 52*(3), 591-620.

Tu, Y., & Lu, X. (2014). Do Ethical Leaders Give Followers the Confidence to Go the Extra Mile? The Moderating Role of Intrinsic Motivation. *Journal of Business Ethics, 135*(1), 129-144. doi:10.1007/s10551-014-2463-6

Xie, Z., Wu, N., Yue, T., Jie, J., Hou, G., & Fu, A. (2020). How Leader-Member Exchange Affects Creative Performance: An Examination From the Perspective of Self-Determination Theory. *Front Psychol, 11*, 573793. doi:10.3389/fpsyg.2020.573793

Yi, L., Uddin, M. A., Das, A. K., Mahmood, M., & Sohel, S. M. (2019). Do Transformational Leaders Engage Employees in Sustainable Innovative Work Behaviour? Perspective from a Developing Country. *Sustainability, 11*(9). doi:10.3390/su11092485

Yidong, T., & Xinxin, L. (2012). How Ethical Leadership Influence Employees’ Innovative Work Behavior: A Perspective of Intrinsic Motivation. *Journal of Business Ethics, 116*(2), 441-455. doi:10.1007/s10551-012-1455-7

Zhang, H., Kwan, H. K., Zhang, X., & Wu, L.-Z. (2012). High Core Self-Evaluators Maintain Creativity. *Journal of Management, 40*(4), 1151-1174. doi:10.1177/0149206312460681

Zhang, X., & Bartol, K. M. (2010). Linking empowering leadership and employee creativity: The influence of psychological empowerment, intrinsic motivation, and creative process engagement. *Academy of management Journal, 53*(1), 107-128.
